# Supplementary material for: Vascular Access for Hemodialysis and Right Ventricular Remodeling: A Prospective Echocardiographic Study
Source: J Clin Med. 2025 Aug 7;14(15):5565. doi: 10.3390/jcm14155565 (PMC12347563; doi:10.3390/jcm14155565)
Supplement: Supplementary file 1 [file jcm-14-05565-s001.zip › jcm-3787974-supplementary.pdf]

Supplementary Table S1. Multivariate predictors of right ventricular dilatation after AVF placement.

Regression variable	Multivariate analysis			
	**β**	**95% CI**	***p*-Value**	**VIF**
Coronary artery disease	0.313	0.014 to 0.400	0.038	1.510
Follow up – OH	0.533	0.029 to 0.113	0.003	1.780
3D RVEDVi	-0.267	-0.01 to 0.001	0.073	1.553
RVFWLS	-0.046	-0.024 to 0.018	0.767	1.912
E/e′	0.283	0.002 to 0.025	0.030	1.112

Multivariate regression analysis was performed with significant predictors in univariate analysis (to avoid excessive collinearity RVFWLS was used instead of RVEF and RVGLS for RV function).
β–standardized regression coefficient; CI–confidence interval; E/e′–ratio of early mitral inflow velocity to early diastolic mitral annular velocity; OH–overhydration (from Body Composition Monitor); RVEDVi–right ventricular end-diastolic volume index; RVFWLS–right ventricular free wall longitudinal strain; VIF–variance inflation factor.
